# Implementation and clinical application of a deformation method for fast simulation of biological tissue formed by fibers and fluid

**DOI:** 10.1186/s13029-016-0054-x

**Published:** 2016-04-15

**Authors:** Ana Gabriella de Oliveira Sardinha, Ceres Nunes de Resende Oyama, Armando de Mendonça Maroja, Ivan F. Costa

**Affiliations:** Faculdade UnB Planaltina, University of Brasilia, 70919-970 Brasilia, DF Brazil; Medicine Department, University of Brasilia, 70919-970 Brasilia, DF Brazil

**Keywords:** Image-guided surgery, Computer-assisted intervention, Soft tissue biomechanics, Real-time interactive simulation, Virtual reality

## Abstract

**Background:**

The aim of this paper is to provide a general discussion, algorithm, and actual working programs of the deformation method for fast simulation of biological tissue formed by fibers and fluid. In order to demonstrate the benefit of the clinical applications software, we successfully used our computational program to deform a 3D breast image acquired from patients, using a 3D scanner, in a real hospital environment.

**Results:**

The method implements a quasi-static solution for elastic global deformations of objects. Each pair of vertices of the surface is connected and defines an elastic fiber. The set of all the elastic fibers defines a mesh of smaller size than the volumetric meshes, allowing for simulation of complex objects with less computational effort. The behavior similar to the stress tensor is obtained by the volume conservation equation that mixes the 3D coordinates. Step by step, we show the computational implementation of this approach.

**Conclusions:**

As an example, a 2D rectangle formed by only 4 vertices is solved and, for this simple geometry, all intermediate results are shown. On the other hand, actual implementations of these ideas in the form of working computer routines are provided for general 3D objects, including a clinical application.

## Background

A realistic and fast soft tissue model must be used effectively in various medical applications, such as planning surgery procedures, image-guided surgery, image registration, diagnosis, biomechanical data refinement, and for training physicians [1].

There are many deformable physics-based methods used for surgical simulation. Meier [2] and Badosgan [3] report on some of the following methods: boundary element method, tensor-mass model, point-associated finite-field approach, and the most widely used finite element method and mass-spring model. They highlight the advantages and drawbacks of each method regarding the level of accuracy, computational load, difficulties and needs during implementation, numerical stability, etc.

The new method presented by Costa [1] runs in real time and can simulate biological soft tissues formed by fluid and a dense network of deformable fibers connecting surface vertices. The deformed state of the mesh is computed equating internal forces, due to fluid pressure and fiber tension, with external forces acting in an area associated with each superficial vertex. The fibrous tissue is similar to mass-spring simulation. However, unlike mass-spring simulation, point masses are not necessary. The mass is distributed in the entire object through fluid density. By enforcing the volume conservation, a behavior similar to stress tensor is obtained, reminiscent of the finite element method. As a result, this method provides some interesting outcomes. It is suited to anisotropic elasticity and non-linear stress–strain relationship. The results are accurate independent of mesh discretization. Only a few material parameters are needed. On the other hand, an important limitation of this method is that it is only valid for objects filled with fluids. Another drawback is that it has no dynamic behavior. Therefore, movements such as waves and vibrations on the object surface cannot be simulated. However, the quasi-static approach has the advantage of being numerically stable.

For problems concerning long-range connections, like the behavior produced by fibers, this approach defines a mesh of smaller size than volumetric meshes, allowing simulation of complex objects with less computational effort. Moreover, user interaction is minimized by dismissing the tedious and time-consuming need for mesh generation [4] and using a fully automated node-fiber-node model instead. On the other hand, volumetric meshes with only local connections can produce a sparse matrix, in which case the numeric solution would be asymptotically faster than this method.

**Fig. 1:**
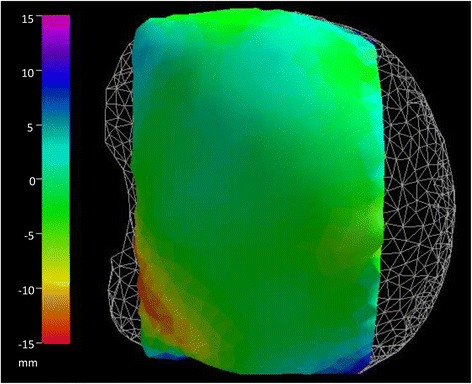
The white mesh represents the un-deformed liver surface. The color code represents the distance between the simulated and the real deformed surface from two vertical compression paddles. All dimensions are in mm. In the red-yellow region, the real surface data discretization is too coarse in order to reproduce the folds. Then, in this region, the distance between the surfaces increased mainly due to data acquisition procedure rather than to the simulation performance

Validation was done by comparing deformation simulation and a real ex vivo bovine liver [1]. A compression was made using two horizontal compression paddles in a form similar to the deformation obtained during a mammographic examination. The results of this comparison show a high degree of similarities between the experimental results and the calculated deformations (Fig. 1). The distance between the simulated and the real deformed surface has a standard deviation of about 1 % of the liver length.

The central theme of this paper is to provide a step by step discussion, algorithmic, and actual implementations in the form of working computer routines of the ideas expressed in Costa [1] for fast simulation of biological tissue. In addition, the software for clinical applications is shown, for the first time, by using the program to deform a 3D breast image of a real patient through the use of a 3D scanner in a hospital environment.

## Methods

### Surface geometry definition

The surface geometry of the objects to be simulated can be derived from data scanned from real data or from intermediate models. In general, this data is given as a set of surface points (vertex) positions.

For a deformable object, a vector ${\overrightarrow{s}}_i$ represents the vertices’ 3D positions, where *i* enumerates each vertex between *1* and the total number of surface points *N*. For the computational code, the x-, y- and z-components of the vector ${\overrightarrow{s}}_i$ are stored in (*X*[*i*], *Y*[*i*], *Z*[*i*]) and *N* is stored in the variable *NVertex.*

The deformation for each vertex can be specified by a displacement vector field ${\overrightarrow{u}}_i$ and its x-, y- and z-components are stored in unique column matrix (*u*[*i*], *u*[*i* + *NVertex*], *u*[*i* + 2 * *NVertex*]).

A triangular mesh comprises a set of *N*′ triangles (in three dimensions) connected by their common vertices and defines the surface shape of an object in 3D solid modeling. To describe each triangle we enumerate three vertices *V[1][i]*, *V[2][i],* and *V[3][i]* which must be connected to form a face, similar to the definition of the WaveFront Object (.obj) File Format [5]. In this case, the index *i* enumerates each triangle between *1* and the total number of surface triangular faces *N’* which in the computational code is stored as *NFaces*. Note that the *V[1][i]*, *V[2][i],* and *V[3][i]* have values between 1 and the total number of surface points *N*.

### Computer routines for faces and vertex areas

The flat nature of triangles makes it simple to determine their normal vector, a three-dimensional vector ${\overrightarrow{A}}_i$ perpendicular to the *i-th* triangle’s surface. Vector ${\overrightarrow{A}}_i$ can be obtained by calculating the cross product between the vectors that form two edges of the triangle divided by two. Thus the modulus of ${\overrightarrow{A}}_i$ is the area of the triangle. The direction of vector ${\overrightarrow{A}}_i$ can be chosen to point outside of the object.

The routine below calculates the vector ${\overrightarrow{A}}_i$. The input variables are vertex vectors ${\overrightarrow{s}}_i$ and the vertices of each triangular face: *V[1][i]*, *V[2][i],* and *V[3][i].* On output, the Cartesian components of the vector ${\overrightarrow{A}}_i$ are stored in variables: *PerpendicularFaceX[i], PerpendicularFaceY[i]* and *PerpendicularFaceZ[i]*, the surface area of each face represented by the modulus $\left|{\overrightarrow{A}}_i\right|$ is stored in variable *PerpendicularFace[i]* and faces total area is stored in variable *AreaFacesTotal*. The faces total area is given by ${\displaystyle {\sum}_{i=1}^{N^{\prime }}\left|{\overrightarrow{A}}_i\right|}$.
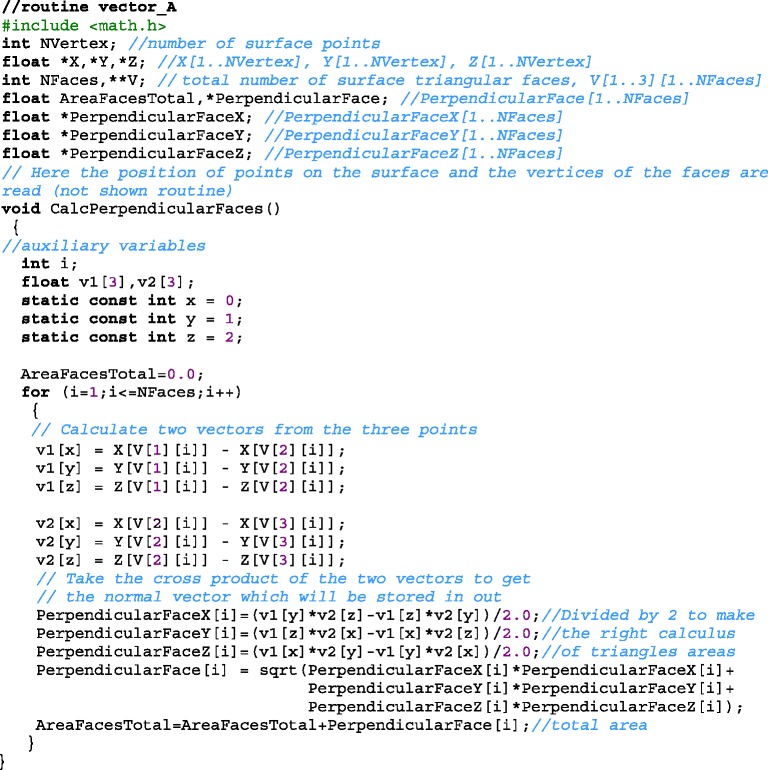


In our method, the deformation of 3D objects is performed through the displacement of its vertices. An area vector is assigned to each vertex, in order to determine the force acting over its surface. This area is defined from the area vectors of the triangles (faces) in the vicinity of the vertex.

The following routine calculates the auxiliary variable *Shared[i][kk]* that stores the identifiers of *kk* triangles (faces) neighbors to a given vertex *i*. The number of neighboring faces of each vertex *i* is stored on the output variable *NFacesSharingVertex[i]*. For example, *Shared[25][2]=40*, it means that the face *40* has been identified as the second triangle adjacent to the vertex *25* and *NFacesSharingVertex[25]=3* informs that there are three neighboring faces to the vertex *25*. The routine has as input variables the vertices *V[1][i]*, *V[2][i],* and *V[3][i]* of each triangular face.
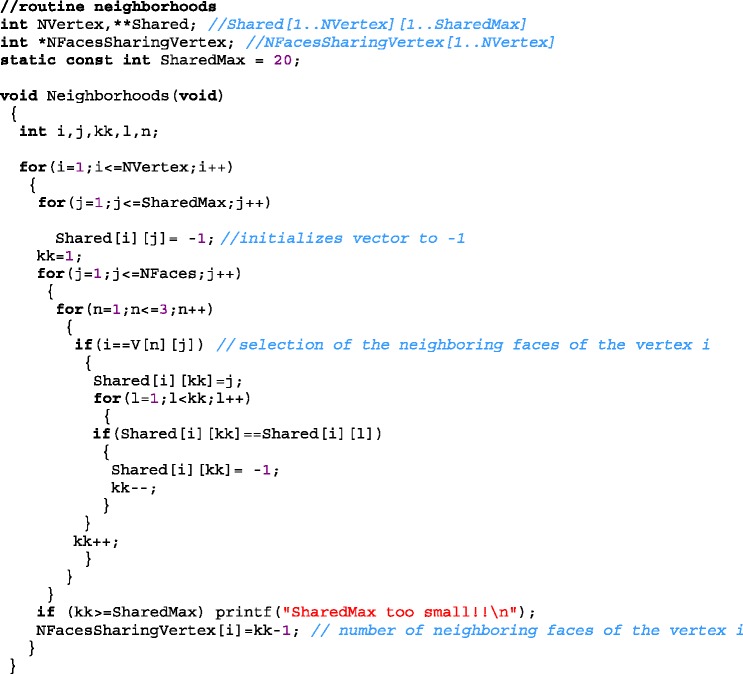


**Fig. 2:**
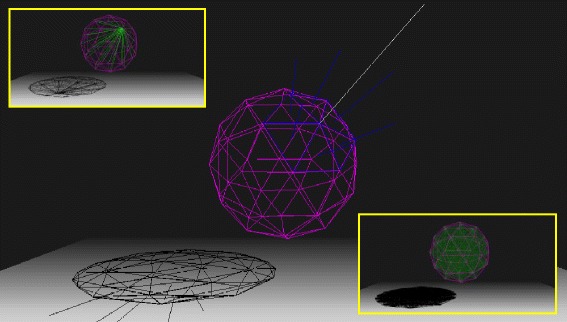
A purple sphere formed by 42 vertices (80 triangles). The blue lines represent the vector area for each triangle neighboring a vertex, for which a blank line represents its resulting vector area. Upper inset: 41 green lines representing the fibers for one vertex. Bottom inset: all superposed fibers (green) for the sphere

Now we can define an area vector ${\overrightarrow{S}}_i$ for each vertex as ${\overrightarrow{S}}_i\equiv C{\displaystyle {\sum}_k}{\overrightarrow{A}}_k$ where *C* is normalization constant, i.e. the area vector for a vertex is proportional to the sum of the area vectors of the triangles in the vicinity of the vertex. *k* values in the summation for each vertex *i* are stored in the variable *Shared[i][k],* where *k* varies from *1* to *NFacesSharingVertex[i].* Figure 2 illustrates these vectors for the case of a sphere. The triangles that form the surface of a sphere are show in purple. Vectors ${\overrightarrow{A}}_k$ are shown as blue lines and ${\overrightarrow{S}}_i$ are represented by a white line for one vertex.

The shape of area $\left|{\overrightarrow{S}}_i\right|$ is not a triangle in general. But the sum of the modulus of areas ${\overrightarrow{A}}_i$ or ${\overrightarrow{S}}_i$ respectively on all faces or vertices must be equal to the solid surface area, i.e.

1 $$ {\displaystyle {\sum}_{i=1}^{N^{\prime }}\left|{\overrightarrow{A}}_i\right|}={\displaystyle {\sum}_{i=1}^{N^{\prime }}\left|{\overrightarrow{S}}_i\right|=\mathrm{surface}\ \mathrm{area}\ \mathrm{of}\ \mathrm{the}\ \mathrm{object}} $$

Then the area of the vertex *i* must be defined as

2 $$ {\overrightarrow{S}}_i\equiv \left(\frac{{\displaystyle {\sum}_{i=1}^{N^{\prime }}}\left|{\overrightarrow{A}}_i\right|}{{\displaystyle {\sum}_{i=1}^N}\left|{\displaystyle {\sum}_k}{\overrightarrow{A}}_k\right|}\right){\displaystyle {\sum}_k{\overrightarrow{A}}_k} $$

The x-, y- and z-components of the vector ${\overrightarrow{S}}_i$ are stored in variables *PerpendicularVertexX[i], PerpendicularVertexY[i] and PerpendicularVertexZ[i]*. The routine vector_S implements Eqs. 1 and 2. From the vector area of faces ${\overrightarrow{A}}_i$ (input) are determined vector area of each vertex ${\overrightarrow{S}}_i$ and the area of the object, stored in variable *AreaVertexTotal* (outputs).
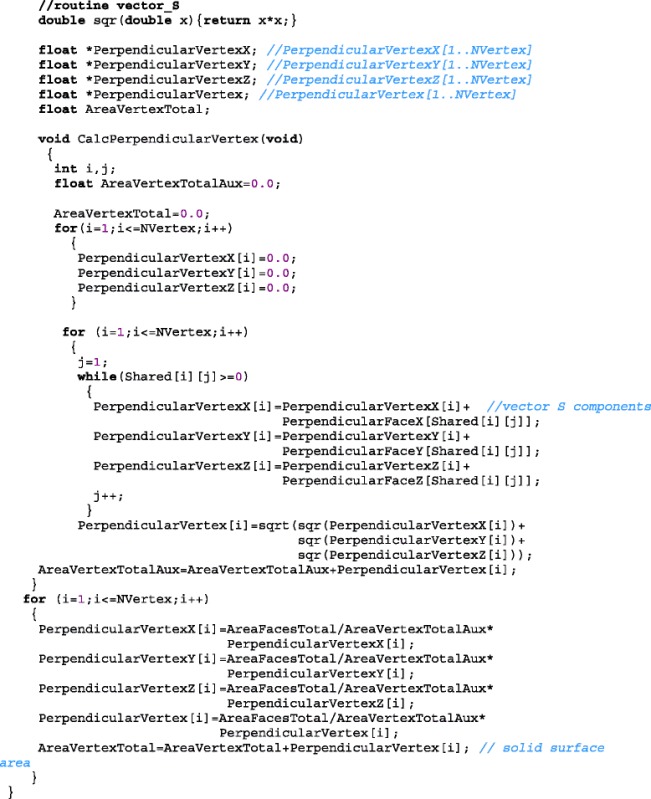


To verify the accuracy of the area calculation and the computer code, a comparison was made between areas of various objects (Platonic solids and sphere) calculated by the equations shown above and by the analytical area calculation. The results are the same for at least four significant figures. Some of these results can be seen in Table 1.

**Table 1:** Surface area of objects which the circumscribed sphere (one that touches the polygon at all vertices) has a unitary radius (*R=1*). The number of vertices *N* and the number of triangular faces *N’* are also shown

Object	*N*	*N’*	Analytical Equation	Analytical Result	${\displaystyle \sum_{i=1}^N\left|{\overrightarrow{S}}_i\right|}$	${\displaystyle \sum_{i=1}^{N^{\prime }}\left|{\overrightarrow{A}}_i\right|}$
Octahedron	6	8	$4\sqrt{3}{R}^2$	6.92820	6.92820	6.92820
Icosahedron	12	20	${\scriptscriptstyle \frac{40\sqrt{3}}{5+\sqrt{5}}}{R}^2$	9.57454	9.57454	9.57454
≈ Sphere	642	1280	≈ 4*πR* ^2^	12.5664	12.5037	12.5065

### Deformation routine

The general result of the Costa deformation method [1] for fast simulation of biological tissue formed by fibers and fluid can be written as a set of *3N +1* variables: *3N* displacements ${\overrightarrow{u}}_1,\ {\overrightarrow{u}}_2,\ \cdots, {\overrightarrow{u}}_i,\cdots, {\overrightarrow{u}}_N$ and a variation of internal pressure *P* (stored in variable *u*[3 * *NVertex* + 1]). The approach implements a quasi-static solution for elastic global deformations of objects filled with fluid and fibers. The static condition states that the internal force on the surface, due to all fibers ${\overrightarrow{F}}_i^{fibers}$ and the force due to the liquid

3 $$ {{\overrightarrow{F}}_i}^{liquid}=P{\overrightarrow{S}}_i $$

have a corresponding external force of the same magnitude but in opposite sense at each point of the object surface. The external forces are due to contact forces ${\overrightarrow{F}}^{contact}$ or forces resulting from accelerations. For the most common situation the acceleration is due to the gravitational field *a*. For this case the pressure is given by *ρh*_*i*_*a* where *h*_*i*_ is the vertical component of the distance from the top to the vertex *i* and *ρ* is the density of the fluid. The result is three equations for each vertex given by

4 $$ {\overrightarrow{F}}_i^{fibers}-P{\overrightarrow{S}}_i={\overrightarrow{F}}_i^{contact}+\rho {h}_ia{\overrightarrow{S}}_i $$

and one equation for the conservation of volume

5 $$ {\displaystyle {\sum}_{i=1}^N{\overrightarrow{S}}_i}\cdot {\overrightarrow{u}}_i=0 $$

Note that Eq. 5 couples the dislocations (and hence, forces) in perpendicular directions (*x*, *y* and *z*). This coupling creates an effect somewhat similar to the stress and strain tensors in standard elastic theory.

The force due to one fiber connecting vertex *i* and *j* obeys Hook’s Law and is proportional to the fiber’s area *(S*_*i*_*+S*_*j*_*)/2* and inverse to its length $\left|{\overrightarrow{s}}_i-{\overrightarrow{s}}_j\right|$, where ${\overrightarrow{s}}_i$ and ${\overrightarrow{s}}_j$ are the positions of vertices *i* and *j*. The force due to all fibers ${\overrightarrow{F}}_i^{fibers}$ is obtained by connecting each vertex *i* to other vertices by a set of *N-1* elastic fibers. These fibers are shown as green lines in the upper inset of Fig. 2 for a sphere.

The process of connecting a vertex to other vertices is repeated for each vertex. This meshing strategy for filling the objects superposes fibers during each connection (bottom inset of Fig. 2). Then the superposed volume depends on the mesh discretization. Therefore, a factor *N-1* must be included in the denominator in order to maintain *Y*_*ij*_ a constant value, independent of the discretization, for bulk fibers:

6 $$ {\overrightarrow{F}}_i^{fibers}={\displaystyle {\sum}_{j\ne i}^N\frac{-{Y}_{ij}\left({S}_i+{S}_j\right)\left({\overrightarrow{u}}_i-{\overrightarrow{u}}_j\right)}{2\left(N-1\right)\left|{\overrightarrow{s}}_i-{\overrightarrow{s}}_j\right|}}. $$

On the other hand, for the surface connections

7 $$ {\overrightarrow{F}}_i^{fibers}={\displaystyle {\sum}_k\frac{-{\gamma}_{ik}\left({S}_i+{S}_k\right)\left({\overrightarrow{u}}_i-{\overrightarrow{u}}_k\right)}{2{\left|{\overrightarrow{s}}_i-{\overrightarrow{s}}_k\right|}^2}} $$

where *Y*_*ij*_ and *γ*_*ik*_ are respectively the force per unit of area and length, whose value can be chosen to be Young’s Modulus and superficial tension or their values must be set in a way to fit an experimental deformation result.

These equations can be written as a problem of type ***A****.****x****=****B*** where ***B*** is the column vector defined by the right hand side of Eq. 4 (${\overrightarrow{F}}_i^{contact}+\rho {h}_ia{\overrightarrow{S}}_i$) and one extra element equal to zero that imposes the conservation of volume (right hand side of Eq. 5). The left hand sides of Eqs. 4 and 5 define the square matrix ***A***.

The resulting vector ***x*** gives all the displacements and the pressure variation. An example of matrices ***x***, ***A*** and ***B*** can be seen in Eq. 8.

### Border conditions

The degenerate first mode, corresponding to the zero eigenvalue, represents a rigid body translation because, although moving, each vertex is stationary relative to the other. Of course, the presence or absence of this degenerate mode will not influence the purely deformable characteristics of the system. This degeneracy can be removed using boundary condition.

For boundary condition some vertices can be considered fixed to an external support. To achieve this condition, vertex *i* must not move, or moves a negligible amount compared to the movement of the others vertices. The resulting movement *x*_*i*_ of the vertex is controlled by the size of element *A*_*ii*_, because each element of the product of matrices ***A*** and ***x*** is naturally expressed as the sum of *N* products *A*_*ij*_*x*_*j*_. In order to impose the border conditions, i.e., make *x*_*i*_*<<x*_*j*_, the element *A*_*ii*_ should be done much bigger than the other elements *A*_*ij*_ in line *i* of matrix ***A***. An example of this procedure can be seen in section "An example for a rectangle".

### Computer routines for general 3D objects

We need to create a matrix *A[1..3N+1] [1..3N+1]* as the input matrix of equation ***A****.****x*** = ***B***. A large number of elements in matrix *A* in general vanish. Thus we initialize by writing zeros in all elements of this matrix.

For a deformable object the triangles’ positions and shapes change. Therefore, we need to recalculate the perpendicular of each triangle and vertex at each calculation step.

Finally, we need to implement Eqs. 3, 4, 5, 6 to 7 and the border conditions. The elastic properties of the object are input data, stored in the variables *Y*_*x*_*, Y*_*y*_ and *Y*_*z*_ (Young’s modulus) and *gamma* (superficial tension). Then, the ***A*** matrix (output) is determined from the quantities determined in the previous routines (inputs): ${\overrightarrow{s}}_i$, ${\overrightarrow{S}}_i$, *Shared[i][kk] and* the vertices *V[1][i]*, *V[2][i],* and *V[3][i]* of each face.
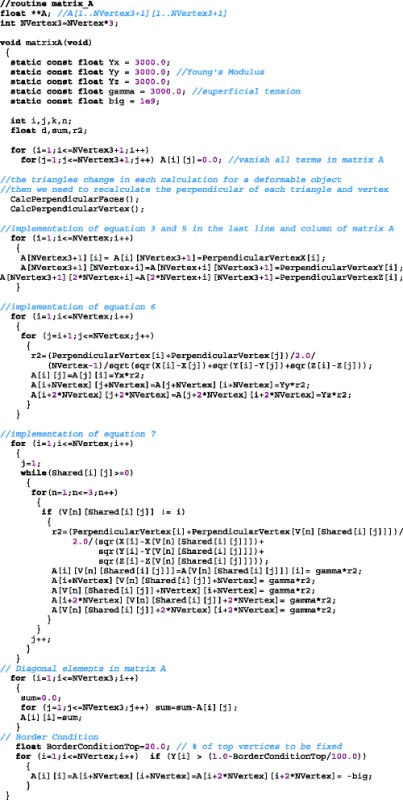


We need to create *B[1..3N+1]* as the input containing the right-hand side of equation ***A****.****x*** = ***B***. As the routine input data has the following constants: the applied force is stored in the variable *Force*, the product gravitational field and the density of the liquid is stored in the variable *gd*; and the number of the vertex where force is applied is stored in the variable *move*. So, we implemented the right side of Eq. 4, creating the vector ***B***.
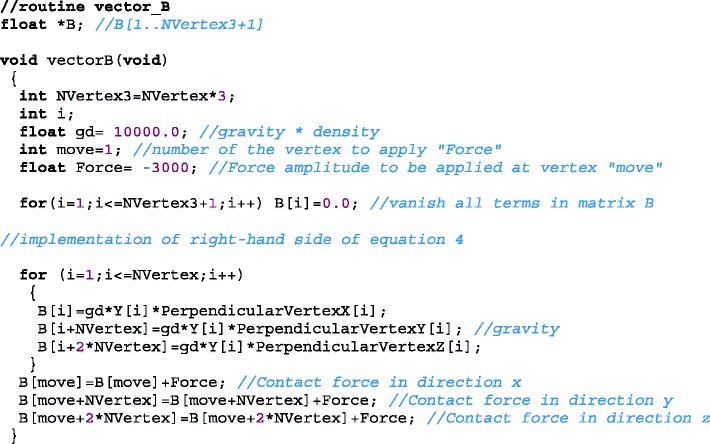


### Concave objects

Note that all fibers effectively exist for a convex object. However for a concave object, some fibers connect pairs of vertices beyond the surface of the object, so these fibers do not actually exist. In order to consider this situation, forces due to fibers need to be removed when they pass through the surface. Then the number *N* is equal to the number of vertices only for the convex object. For concave object, *N* must be equal to the number of effective connections.

Two tests must be done in order to detect the fibers that pass through the surface of the object. First, we need to test if the fiber starting at vertex *i* goes inside or outside the object. Then, we calculated the scalar product for all *k* triangles neighboring the vertex *i:*${\overrightarrow{A}}_k\cdot \left({\overrightarrow{S}}_i-{\overrightarrow{S}}_j\right)$. If there is any negative result, the fiber connecting the vertices *i* and *j* goes outside the object and the matrix element *A*_*ij*_ must be set equal to zero.

A second test is needed because if a fiber intersects any triangle, it goes outside the object. Then we used the *fast 3D line segment-triangle intersection test* developed by Chirkov [6]. If a fiber that connects the vertices *i* and *j* intersects any triangle, the matrix element *A*_*ij*_ also must vanish. In this link [7] there is an executable beta version of our software with this and other functionalities.

### The matrix solution

The routine *vector_A*, *neighborhoods* and *vector_S* must be calculated only once, before solving the routines *matrix_A* and *vector_B* for each deformation step. Therefore, the initial routines sequence can be
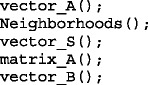


To simulate a non-linear stress–strain relation the value of *Y*_*ij*_ can be defined as a function of the displacement ${\overrightarrow{u}}_i-{\overrightarrow{u}}_j$ in the last step. Shortly after each increment of deformation, the element geometry and stiffness value is updated to model the nonlinear behavior of the material. The variation of external force on each interaction should be chosen to obtain the desired precision or an acceptable calculation time. Therefore, in this case, matrix *A* must be recalculated at each step.

But for situations where the area ${\overrightarrow{S}}_i$ and the distance of each vertex $\left|{\overrightarrow{s}}_i-{\overrightarrow{s}}_j\right|$ varies in a negligible amount on the left hand side of Eqs. 4 and 5 and the elasticity is linear (surface tension γ_ik_ and Young’s Modulus *Y*_*ij*_ are constants independent of the deformation), matrix ***A*** is constant and can be operated once. For example, LU decomposition from the book Numerical Recipes in C [8] was used to solve the problem ***A.x=B***. If matrix A is constant, the routine given in this book allows LU decomposition result to be left in place for successive calls with different right-hand sides ***B*** to achieve a greatly reduced calculation time.

## Results and discussions

### An example for a rectangle

**Fig. 3:**
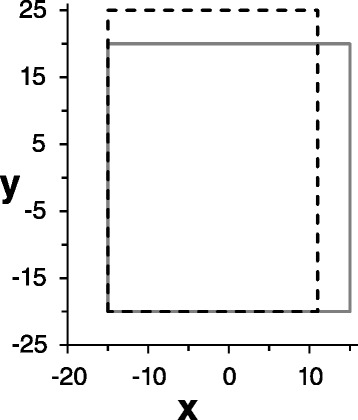
Un-deformed rectangle is shown in solid line. The lower left corner is fixed, the lower right corner is constrained to move only horizontally. The dashed line shows the new shape after forces (numeric of 1680) are applied to the upper corners vertically upward

Let us consider the problem of a rectangle with edges ± 15*î* ± 20*ĵ* that defines the four vertices ${\overrightarrow{s}}_i$ (Fig. 3). For this first example, the units are arbitrary and simplicity surface tension effects are not taken into account. In this case *N=N’=4* and the “areas” *A*_*i*_ in this two-dimensional case are actually the edges of length *30* and *40*. Each vertex has two neighbors, one in *x* direction and another in the direction *y*. Then using Eq. 1 and 2 we obtain that ${\overrightarrow{S}}_i=7\left(\pm 4\widehat{i}\pm 3\widehat{j}\right)$ and $\left|{\overrightarrow{S}}_i\right|=35$.

For simplicity, the 2D Young’s Modulus is set to be a constant *Y*_*ij*_*=360* and no acceleration effect is taken into account. The boundary conditions are chosen to be as least restrictive as possible: the lower left corner is fixed, the lower right corner is constrained to move only horizontally. In order to obtain these boundary conditions, we make the matrix elements *A*_*ii*_ large, equal to *10*^*9*^, for these vertices in these directions.

Forces of equal magnitude *F*_*i*_^*contact*^*=1680* are applied to the upper corners vertically upward. Using Eq. 4 and 5 for this rectangle, we can write the problem ***A****.****x*** = ***B*** as:

8 $$ \left[\begin{array}{ccccccccc}\hfill 329\hfill & \hfill -105\hfill & \hfill -84\hfill & \hfill -140\hfill & \hfill 0\hfill & \hfill 0\hfill & \hfill 0\hfill & \hfill 0\hfill & \hfill 28\hfill \\ {}\hfill -105\hfill & \hfill 329\hfill & \hfill -140\hfill & \hfill -84\hfill & \hfill 0\hfill & \hfill 0\hfill & \hfill 0\hfill & \hfill 0\hfill & \hfill 28\hfill \\ {}\hfill -84\hfill & \hfill -140\hfill & \hfill {10}^9\hfill & \hfill -105\hfill & \hfill 0\hfill & \hfill 0\hfill & \hfill 0\hfill & \hfill 0\hfill & \hfill -28\hfill \\ {}\hfill -140\hfill & \hfill -84\hfill & \hfill -105\hfill & \hfill 329\hfill & \hfill 0\hfill & \hfill 0\hfill & \hfill 0\hfill & \hfill 0\hfill & \hfill -28\hfill \\ {}\hfill 0\hfill & \hfill 0\hfill & \hfill 0\hfill & \hfill 0\hfill & \hfill 329\hfill & \hfill -105\hfill & \hfill -84\hfill & \hfill -140\hfill & \hfill 21\hfill \\ {}\hfill 0\hfill & \hfill 0\hfill & \hfill 0\hfill & \hfill 0\hfill & \hfill -105\hfill & \hfill {10}^9\hfill & \hfill -140\hfill & \hfill -84\hfill & \hfill -21\hfill \\ {}\hfill 0\hfill & \hfill 0\hfill & \hfill 0\hfill & \hfill 0\hfill & \hfill -84\hfill & \hfill -140\hfill & \hfill {10}^9\hfill & \hfill -105\hfill & \hfill -21\hfill \\ {}\hfill 0\hfill & \hfill 0\hfill & \hfill 0\hfill & \hfill 0\hfill & \hfill -140\hfill & \hfill -84\hfill & \hfill -105\hfill & \hfill 329\hfill & \hfill 21\hfill \\ {}\hfill 28\hfill & \hfill 28\hfill & \hfill -28\hfill & \hfill -28\hfill & \hfill 21\hfill & \hfill -21\hfill & \hfill -21\hfill & \hfill 21\hfill & \hfill 0\hfill \end{array}\right]\left[\begin{array}{c}\hfill {u}_{1x}\hfill \\ {}\hfill {u}_{2x}\hfill \\ {}\hfill {u}_{3x}\hfill \\ {}\hfill {u}_{4x}\hfill \\ {}\hfill \begin{array}{c}\hfill {u}_{1y}\hfill \\ {}\hfill {u}_{2y}\hfill \\ {}\hfill {u}_{3y}\hfill \\ {}\hfill {u}_{4y}\hfill \end{array}\hfill \\ {}\hfill P\hfill \end{array}\right]=\left[\begin{array}{c}\hfill 0\hfill \\ {}\hfill 0\hfill \\ {}\hfill 0\hfill \\ {}\hfill 0\hfill \\ {}\hfill \begin{array}{c}\hfill 1680\hfill \\ {}\hfill 0\hfill \\ {}\hfill 0\hfill \\ {}\hfill 1680\hfill \end{array}\hfill \\ {}\hfill 0\hfill \end{array}\right] $$

Solving this matrix equation for *u* we obtain the dislocations that should be added to the vertices position to get the new deformed shape. The result for the position of the right corners is *s*_*x*_*=11* and for the upper corners *s*_*y*_*=25*, as shown in Fig. 3. Other examples for 3D objects are shown above.

### An example for a sphere

Let us now consider that a sphere with a radius of one meter. The border conditions impose that all upper vertices, defining a spherical cap of height *0.2*, cannot move. The parameters are defined as: the surface tension *γ*_*ik*_ and Young’s Modulus *Y*_*ij*_ are adjusted to be equal to *3* with units in *kNewtons* and meters; the acceleration due to gravity is acting down and increases at each iteration *1 m/s*^*2*^ in a total of ten steps until the final value *g=10 m/s*^*2*^ is reached; the matrix ***A*** is constant; the number of vertices is *N=162*; and the density of the fluid is *ρ* = 1000 *kg*/*m*^3^. To show the effect of contact forces ${\overrightarrow{F}}^{contact}$*,* at the final step a force $\overrightarrow{F}=-300\left(\widehat{i}+\widehat{j}+\widehat{k}\right)$ is applied to the vertex number *1*.

**Fig. 4:**
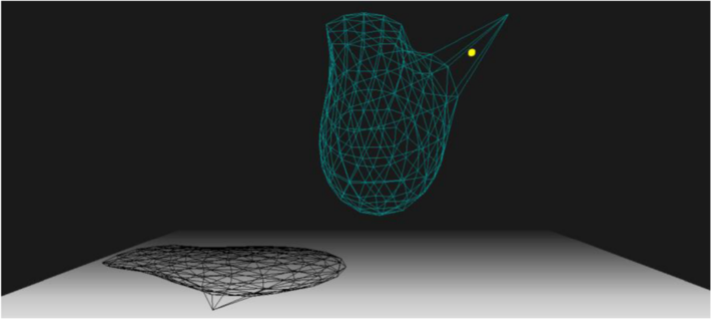
A sphere formed by 320 faces is deformed by the gravitational field and by a vertex pulled in the up right direction

Inside the link [9], we provide a complete source code for a deformable 3D sphere. The result of this code can be seen in Fig. 4.

The ideas given by Wright [10] were used to draw the objects and shadows. In addition, the routine to make spheres was taken from Shreiner [11].

### An example for a real in vivo breast

**Fig. 5:**
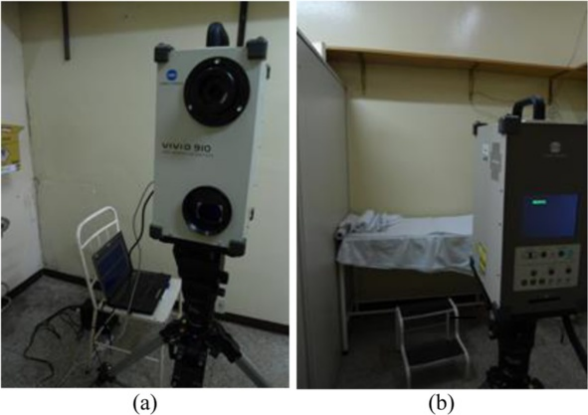
The experimental arrangement in real hospital environment showing the 3D scanner: (**a**) from the patient’s point of view and (**b**) from the 3D scanner operator’s point of view

In order to show the benefit of our software for clinical applications, we used our computer program to deform 3D breasts images, acquired from a set of four patients in a real hospital environment. The 3D geometries of the breasts’ surfaces were obtained using a non-contact 3D Digitizer Konica Minolta Vivid 910, as shown in Fig. 5.

**Fig. 6:**
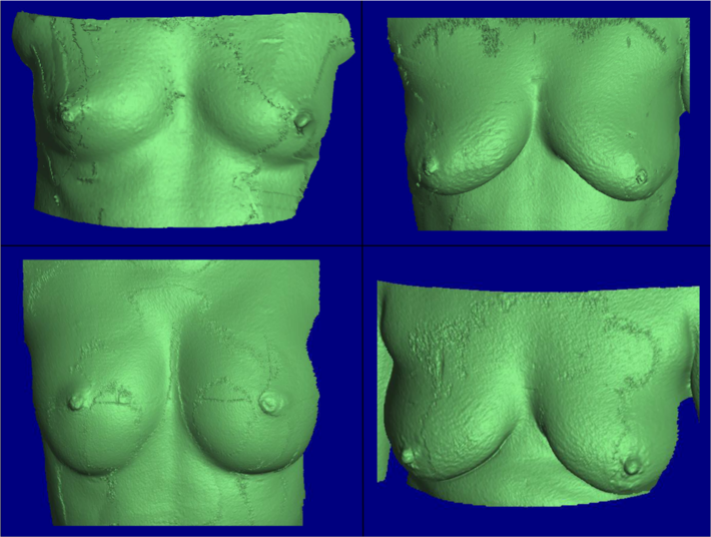
Geometry reconstruction for four real patients breast surfaces

Reconstruction was made by merging different views of the patients in a standing position taken from different angles. Several reference points were located on each patient, in order for an accurate merge. These images were mapped and the final reconstruction can be seen in Fig. 6. These surfaces were exported in obj format and run through our simulator.

The tissues were simulated as isotropic and linear, allowing us to do LU decomposition of the matrix ***A*** only once before the first iteration. Thus, for each interaction the right hand of Eq. 4 (vector ***B***) are updated and new values for ${h}_i,\kern0.49em a\kern0.37em \mathrm{and}\ {\overrightarrow{S}}_i$ must be incorporated at the next calculation step. We choose this procedure because, according to Costa [1], the resultant deformation in this case is similar to the deformation of the slower procedure that repeats LU decomposition at each interaction (updating ${\overrightarrow{S}}_i$ and $\left|{\overrightarrow{r}}_i-{\overrightarrow{r}}_j\right|$).

Nevertheless, for nonlinear approaches the matrix ***A*** changes for each step and it must be recalculated for each iteration, imposing the necessity of the slower method. An example of a nonlinear elasticity is shown in Costa [1].

Best accuracy is expected if small steps are used (great number of interactions), since in this case the variation of ${h}_i,\kern0.49em a\kern0.37em \mathrm{and}\ {\overrightarrow{S}}_i$ is smaller and the outcome get closer to the continuous (analytical) result.

We chose the surface tension *γ*_*ik*_ = 48 *MN*/*m*, the Young’s Modulus *Y*_*ij*_ = 48 *kPa* and the density of the fluid *ρ* = 1000 *kg*/*m*^3^. The boundary conditions in the case of breast deformation are the chest region that restrains the tissue movement in any direction.

**Fig. 7:**
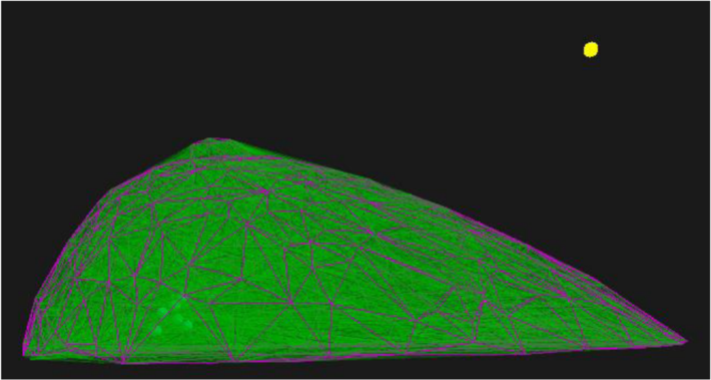
The breast surface with 402 triangles (purple) and the simulated fibers set (green) for patient number four

Figure 7 shows the result for patient number four. The surface of the breast is formed by 402 faces and the simulated fibers are also shown.

**Fig. 8:**
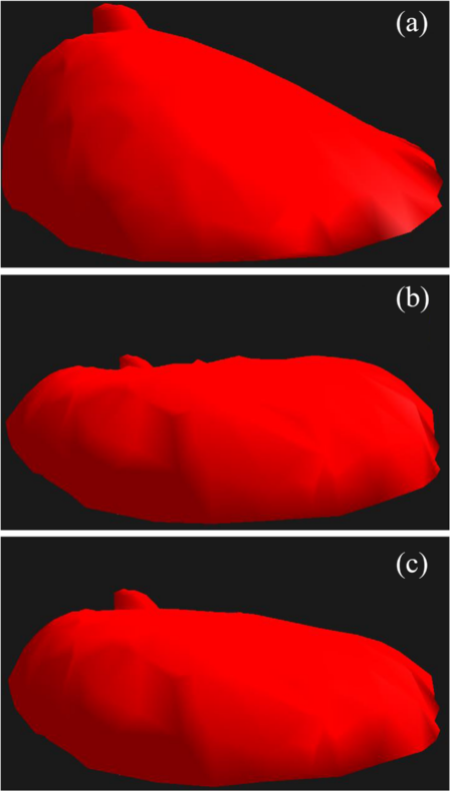
Uncompressed (**a**) and compressed breast using one step (**b**) and 100 steps (**c**) to achieve the total compression field

A compression, perpendicular to the chest plane, was set to the simulated breasts due to the increase of the gravitational field (*10 m/s*^*2*^). The number of steps *n* to achieve the final gravitational field value could vary. The uncompressed (a) and compressed breast shape for the number of steps *n* equal to one (b) and *100* (c) are shown in Fig. 8 for patient number four.

Finally, we decompressed the breast by decreasing the acceleration applied to the deformed breast until the acceleration vanished. The same number of steps used during the compression procedure was used for the decompression. Then the mean difference in the initial (uncompressed) and final (decompressed) nodes position was calculated as:

9 $$ \frac{{\displaystyle {\sum}_{i=1}^N}\left|{\overrightarrow{u}}_i\right|}{N\sqrt{{\left({s}_x^{max}-{s}_x^{min}\right)}^2+{\left({s}_y^{max}-{s}_y^{min}\right)}^2+{\left({s}_z^{max}-{s}_z^{min}\right)}^2}} $$

where *s*_*x*_^*max*^ and *s*_*x*_^*min*^ are the maximum and minimum values of *s*_*x*_ (the same for the directions *y* and *z*).

For a continuous (analytical) approach, no difference on uncompressed and decompressed position are expected, and the result of Eq. 9 must be zero. For a numerical (discrete) approach differences on the initial and final position are expected due to errors introduced in the calculation of matrix ***B*** in each step. However, in our algorithm the results of Eq. 9 are small; numerically *3 %* for *1* interaction and decreased to about *0.3* % and *0.03 %* for *10* and *100* interactions, respectively. And the method evaluation results were similar for all four patients. These results demonstrate a good algorithmic behavior for the complete compression and decompression process of the breasts.

## Conclusions

This work presents the implementation of a new general approach for modeling soft tissue compression process. The method was successfully applied to compress a 3D breast model from real patients.

The fast simulation of deformation of biomaterial using this algorithm could provide more realistic images that could serve for educational, clinical application, and research purposes. The latter include investigations of different breast imaging techniques involving compressed and uncompressed breasts.
